# The Role of High-Frequency Wall Vibrations in Adverse Vascular Remodeling of Arteriovenous Fistula for Hemodialysis

**DOI:** 10.34067/KID.0000001112

**Published:** 2026-01-14

**Authors:** Luca Soliveri, Sofia Poloni, Paolo Brambilla, Simona Zerbi, Giulia Cabrini, Anna Caroli, Andrea Remuzzi, Kristian Valen-Sendstad, Michela Bozzetto

**Affiliations:** 1 Department of Bioengineering, Istituto di Ricerche Farmacologiche Mario Negri IRCCS, Bergamo, Italy; 2 Department of Engineering and Applied Sciences, University of Bergamo, Bergamo, Italy; 3 Diagnostic Radiology, ASST Papa Giovanni XXIII Hospital, Bergamo, Italy; 4 School of Medicine, University of Milano-Bicocca, Milan, Italy; 5 Nephrology and Dialysis Unit, ASST Papa Giovanni XXIII Hospital, Bergamo, Italy; 6 Department of Management, Information and Production Engineering, University of Bergamo, Bergamo, Italy; 7 Department of Computational Physiology, Simula Research Laboratory, Oslo, Norway

**Keywords:** access blood flow, arteriovenous fistula, cardiovascular disease, CKD, dialysis access, hemodialysis access, vascular access, biomarkers, imaging

## Abstract

**Key Points:**

- Flow-induced high-frequency vascular vibrations, computed through fluid-structure interaction simulations, are associated with fistula complications.
- The association between vibrations and arteriovenous fistula complications suggests a potential new mechanism responsible for vascular remodeling.
- The frequency and amplitude of vibrations are potential biomarkers for arteriovenous fistula surveillance.

**Background:**

Despite progress in research, the mechanobiological mechanisms behind adverse vascular remodeling and failure in arteriovenous fistula (AVF) for hemodialysis remain unclear. The aim of this investigation was to assess the association between flow-induced vascular wall vibrations and adverse vascular remodeling in AVFs.

**Methods:**

Six ESKD patients with native distal radio-cephalic AVF were monitored for 1 year with magnetic resonance imaging and Doppler ultrasound examinations. Patients were divided based on AVF outcomes: two maintained proper AVF patency and four developed complications (two venous stenoses and two excessive dilatations). Patient-specific fluid-structure interaction simulations were performed at different time points.

**Results:**

Before vascular remodeling, stenotic AVFs exhibited two dominant frequency bands, between 45 and 100 Hz, while excessively dilated AVFs exhibited a single band at 50 Hz. Before the onset of remodeling, patients with complications exhibited significantly higher vibration amplitude (22.5±5.8 *μ*m versus 6.6±2.0 *μ*m, *P* < 0.01) and high-pass strain ([1.30±0.35] 10^−3^ versus [0.30±0.10] 10^−3^, *P* < 0.01) than those with proper patency. Significant differences in vibration amplitude and high-pass strain were observed between patients with proper patency and those with stenosis (*P* < 0.001 and *P* < 0.01, respectively), and in high-pass strain between patients with preserved patency and those with excessive dilatation (*P* < 0.01).

**Conclusions:**

Specific vibration frequencies and amplitude levels appear to be associated with distinct types of vascular remodeling, indicating that they could potentially be biomarkers for AVF surveillance.

## Visual Abstract

**Figure:**
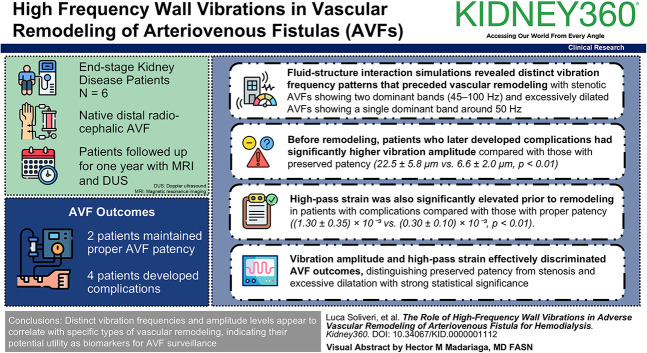

## Introduction

Native arteriovenous fistula (AVF) is the preferred vascular access for hemodialysis,^1,2^ although it has a failure rate of 40% within the first year after surgery.^3–5^ The primary cause of failure is inward vascular remodeling due to intimal hyperplasia, a proinflammatory response promoting smooth muscle cell activation, proliferation, and migration in the intimal layer.^6–12^ Moreover, excessive outward remodeling may also be associated with complications, potentially causing high flow leading to cardiac overload.^2,13^

In the last decades, medical image-based computational fluid dynamics^14–19^ and fluid-structure interaction (FSI)^20–24^ simulations have been used to investigate the abnormal blood flow conditions that arise after the creation of an arteriovenous shunt. Specifically, most research has focused on abnormal wall shear stress (WSS) on the endothelium and its role in adverse vascular remodeling. As a result, various WSS-based indices have been proposed to correlate with AVF failure. In this context, the most recent work from the Hemodialysis Fistula Maturation Consortium demonstrated that higher WSS was positively associated with subsequent lumen expansion and unassisted clinical maturation.^25^ However, despite the increased understanding gained from these studies on the hemodynamic characterization of AVFs, a clear correlation between these indices and pathologic vascular remodeling has not yet been established,^26^ limiting the effectiveness of using WSS as a biomarker or predictor of disease progression in clinical contexts.

Interestingly, recent FSI studies on patient-specific AVF models from our group revealed the occurrence of flow-induced vascular wall vibrations in the AVF vein,^27,28^ suggesting that these vibrations may play a role in adverse vascular remodeling.^29^ In addition, we showed that AVFs characterized by significant adverse vascular remodeling exhibited high-frequency components in AVF sounds recorded with a stethoscope.^30,31^ We have also recently reviewed the existing literature on the biologic effects of wall vibrations and shown that high-frequency mechanical stimuli may influence vascular cells' mechanobiology, leading to both morphologic and functional changes.^32^

Therefore, the current work aimed to investigate the effects of flow-induced vascular wall vibrations on vascular remodeling in six patients with native distal radiocephalic AVFs over a 1-year follow-up period after surgical creation.

## Methods

This report describes six patients with ESKD (five men and one woman; 54±20 years) who underwent native radio-cephalic AVF creation in the forearm and were followed in routine clinical practice at the Nephrology and Dialysis Unit of ASST Papa Giovanni XXIII. Patients with a previously failed native AVF or graft in the same arm were not included. All collected clinical data were fully anonymized and analyzed retrospectively. Clinical characteristics of the patients are summarized in Table 1.

**Table 1:** Clinical data for the six patients involved

Patient information	P1	P2	S1	S2	D1	D2
Weight (kg)	78	67	68	93	77	66
Height (cm)	170	162	170	163	180	173
Native kidney disease	Unknown	ANCA vasculitis	ADPKD	Hypertensive nephropathy	Unknown	IgA nephropathy
Office BP (mm Hg)	150/70	160/90	140/85	125/68	130/85	120/77
Hypertension	Yes	Yes	Yes	Yes	Yes	No
Diabetes	Yes	No	No	No	No	No
Cardiovascular disease	No	No	No	No	No	No
Dialysis modalities at AVF surgery	None	Hemodialysis with CVC	None	None	None	Peritoneal dialysis
AVF access	Left distal radio-cephalic	Left distal radio-cephalic	Left distal radio-cephalic	Left distal radio-cephalic	Right distal radio-cephalic	Left distal radio-cephalic
AVF anastomosis	L-T	L-T	L-T	L-T	L-T	L-T
Time to successful dialysis initiation (d)	33	50	130	43	50	48
BA diameter preoperatively (mm)	5.2	4.3	4.7	5.3	4.8	4.6
RA diameter preoperatively (mm)	3.3	2.4	3.0	2.6	2.4	2.2
CV diameter preoperatively (mm)	3.9	2.8	2.3	2.7	3.0	2.5
BA blood flow preoperatively (ml/min)	60	49	20	103	42	32
RA blood flow preoperatively (ml/min)	37	23	9	55	22	24
BA diameter 1 yr (mm)	5.9	6.6	7.1	6.5	7.1	6.8
RA_prox_ diameter 1 yr (mm)	4.4	6.0	7.0	5.2	5.5	5.4
RA_dist_ diameter 1 yr (mm)	3.2	4.8	2.5	3.6	4.2	4.2
CV diameter 1 yr (mm)	7.2	8.4	2.0	3.5	14.8	13.1
BA blood flow 1 yr (ml/min)	527	840	860	777	1545	801
RA_prox_ blood flow 1 yr (ml/min)	316	568	520	560	916	582
RA_dist_ blood flow 1 yr (ml/min)	27	217	4	91	250	112
AVF outcome	Patent	Patent	Stenosis	Stenosis	Excessive dilatation	Excessive dilatation

ADPKD, autosomal dominant polycystic kidney disease; ANCA, antineutrophil cytoplasmic antibody; AVF, arteriovenous fistula; BA, brachial artery; CV, cephalic vein; CVC, central venous catheter; D, dilatation; L-T, latero-terminal; P, patency; RA, radial artery; S, stenosis.

**Figure 1:**
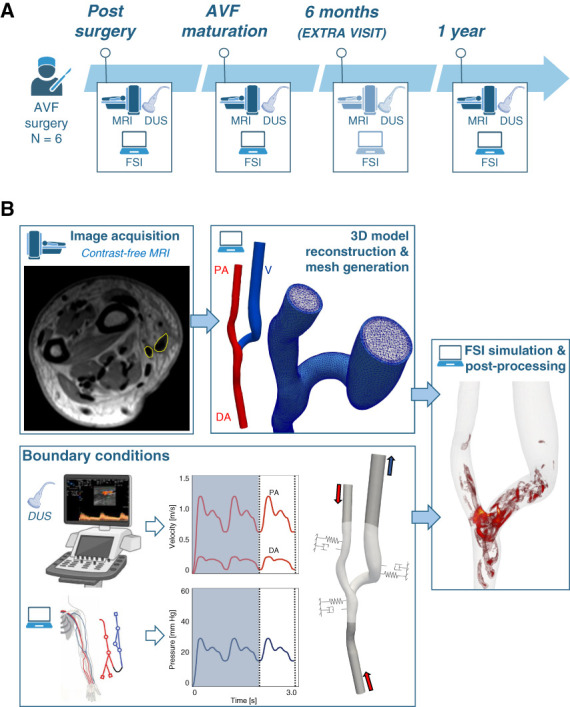
**Investigation timeline and graphical overview of the pipeline.** (A) Investigation timeline showing the time points at which clinical data acquisition and computational analysis were performed. (B) FSI pipeline used to characterize AVF hemodynamics and high-frequency vascular vibrations. 3D, three-dimensional; AVF, arteriovenous fistula; DA, distal artery; DUS, Doppler ultrasound; FSI, fluid-structure interaction; MRI, magnetic resonance imaging; PA, proximal artery; V, vein.

Magnetic resonance imaging (MRI), Doppler ultrasound (DUS), and FSI simulations were performed at three time points: within 3 days of AVF creation, at AVF maturation (around 6 weeks post-surgery), and 1 year post-surgery. For two patients, additional MRI and DUS data were collected at 6 months post-surgery (Figure 1A).

For the analysis of the FSI results, patients were retrospectively divided into three groups based on AVF outcome, assessed by clinical evaluation, which included DUS assessments and any reported AVF complications. Specifically, the AVF outcomes considered were proper patency (P) maintained over time, stenosis (S) formation, and excessive dilatation (D), defined as a cephalic vein diameter exceeding twice the recommended^2^ diameter of 6 mm. Computed hemodynamic patterns and vascular wall vibrations were analyzed in relation to vascular wall remodeling and AVF outcomes over time.

### Medical Imaging Acquisition

The complete FSI pipeline is illustrated in Figure 1B. Non–contrast-enhanced MRI were acquired using a 1.5T scanner, covering an arm region extending approximately 5 cm above and 3 cm below the anastomosis. A detailed description of the MRI protocol can be found in our previous publications.^33–35^ Before each MRI scan, a DUS examination of the AVF was performed to measure vessel diameters and blood flow velocities in the brachial and radial arteries.

### FSI Simulations

MRI-based three-dimensional AVF surfaces were used to generate meshes consisting of approximately 200,000 tetrahedral elements, building upon a previous mesh refinement study.^28^ FSI simulations of the blood flow were performed using turtleFSI solver,^36^ including compliant vascular wall and perivascular tissue modeling. A simulation timestep of 0.1 ms was selected to accurately capture high-frequency fluctuations in velocity and pressure.^37^ Patient-specific flow waveforms obtained from DUS measurements were imposed at the inlet of the proximal and distal radial artery. The simulations accounted for the distinct mechanical properties of the vein and artery, as well as the stiffening and thickening of the cephalic vein during maturation.^38^ The viscoelastic effect of perivascular tissue was modeled with Robin boundary conditions.^39^ For further details on the FSI pipeline, please refer to Supplemental Material and our recent publication.^28^

### Postprocessing of the Results

The *Q*-Criterion was used to highlight vortices and flow instabilities.^28^ Fluid velocity and wall displacement spectrograms were generated to illustrate the evolution of high-frequency content over the cardiac cycle.^40,41^ Vibration and high-pass displacement amplitudes were calculated by high-pass filtering the vascular wall deformations over 25 Hz.^28^ The pointwise 99th spatial percentile^41^ of vibration was used to represent the vibration amplitude progression over time.

Cross-sectional slices of time-averaged vibration amplitude and high-pass strain surface maps were extracted at the time point preceding the main vascular remodeling event (*t*-1; *e.g*., stenosis formation or excessive dilatation) to investigate how these metrics influenced subsequent vascular remodeling at the following timestep. Slices were taken perpendicular to the model's centerlines within the first 2.5 cm of the juxta-anastomotic vein (JAV), with a spacing of 0.1 mm. The average value of each metric was obtained for each slice and considered for statistical analysis, resulting in 25 values per patient.

The average values of common WSS indices in the JAV—time-averaged WSS (TAWSS), oscillatory shear index (OSI), and spectral power index (SPI), a proposed metric of cycle-invariant turbulent-like flow^42^—were computed from FSI simulations to explore their relationship with wall vibrations.

### Statistical Analysis

Statistical analysis was conducted using *R* software (version 4.1.0). Values of vibration amplitude and high-pass strain at time *t*−1 were considered for the analysis to assess their impact on subsequent vascular remodeling. Boxplots were generated to compare the distribution of vibration amplitude and high-pass strain in patients with proper patency and in those experiencing complications. A linear mixed-effects model was fitted using the *lmer* function from the *lme4* package, with “Vibration amplitude” and “High-pass strain” as response variables, Group (Patency, Stenosis, and Dilatation) as fixed effect, and Patient as random effect to account for repeated measures within subjects. A Shapiro-Wilk test was performed to assess the normality of the residuals. Where there was a violation of the normality assumptions, a generalized linear mixed model was subsequently fitted using the *glmmTMB* package, with a Gamma distribution and a log link function. The significance level was set at 0.05 when examining differences between patients with proper patency and those with complications (Patency versus Adverse Remodeling), while for comparisons among the three groups (Patency, Stenosis, and Dilatation), the Bonferroni correction was applied, adjusting the significance level to $\alpha =\frac{0.05}{3}=0.0167$ to account for multiple testing.

Finally, we explore the correlation between WSS indices (TAWSS, OSI, SPI) and wall vibrations and high-pass strain using linear mixed-effects models. Patient identity was included as a random effect to account for repeated measures within subject. The relationships were also evaluated after log_10_-transformation of vibration metrics to account for potential nonlinear scaling. The conditional correlation coefficient (*R*) and significance levels were calculated, and the results were visualized using scatter plots with the model regression line.

## Results

### Evolution in AVF Morphology and Blood Flow versus AVF Outcome

**Figure 2:**
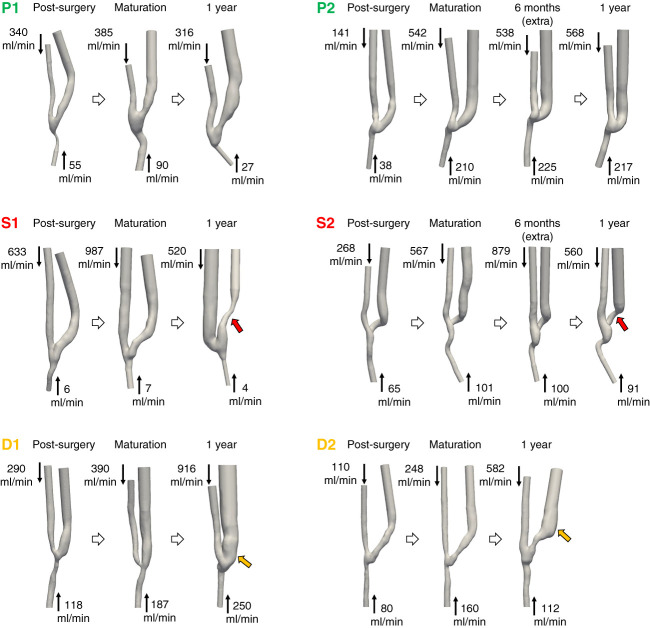
**3D AVF models generated from medical imaging data, with average inlet blood flow rates measured during Doppler ultrasound examinations, for the six AVFs under study, at various time points.** Black arrows indicate the direction of blood flow entering the proximal and distal arteries, while red and orange arrows denote locations of stenosis and excessive dilatation, respectively. D, excessive dilatation; P, patency; S, stenosis.

**Figure 3:**
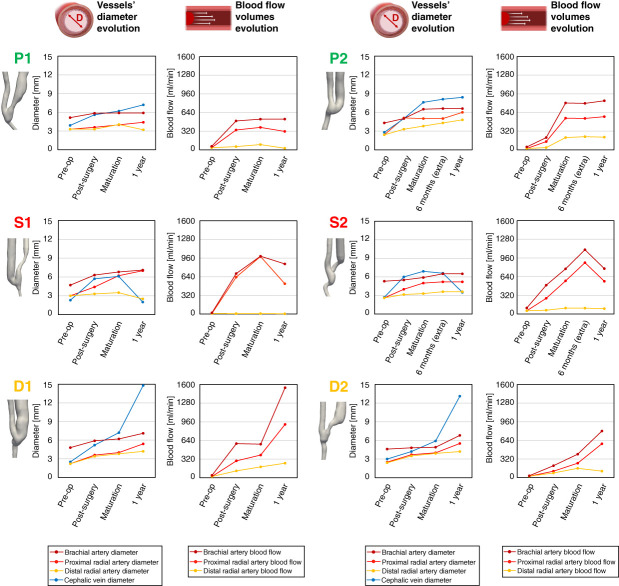
**Evolution over time of brachial artery, radial artery (proximal and distal), and cephalic vein diameters in the six AVFs under study.** The diameters of the radial artery and cephalic vein were measured approximately 2 cm from the anastomosis. The figure also illustrates blood flow volumes entering the vascular access through the brachial artery as well as through the proximal and distal radial arteries.

The six AVFs followed in this investigation had different clinical evolutions and underwent diverse types of remodeling (see Figure 2). The preoperative diameters of all of the radial arteries and cephalic veins were >2 mm, which is the suggested threshold for creating a proper vascular access.^2^ In all patients, the creation of the arteriovenous shunt resulted in a sudden increase in blood flow rates and vessel diameter, particularly of the cephalic vein, already 3 days after surgery, compared with preoperative measurements. All AVFs then experienced proper maturation, achieving a cephalic vein diameter of at least 6 mm, sufficient for successful cannulation,^2^ and a blood flow rate adequate for initiating hemodialysis (see Figure 3).

Patients P1 and P2 maintained AVF patency at the 1-year follow-up, with cephalic vein diameters remaining almost unchanged (7.2 mm and 8.4 mm at 1 year, respectively) and stable blood flow in the brachial artery over time (cycle-averaged blood flow volumes of 527 ml/min for P1 and 840 ml/min for P2 at 1 year), enabling them to continue hemodialysis. By contrast, patients S1 and S2 developed stenosis in the JAV, leading to significant narrowing of the cephalic vein, with diameters returning approximately to preoperative values (minimum diameters of 2.0 mm and 3.5 mm, respectively, at 1 year) and a decrease in brachial blood flow rate (from 992 to 860 ml/min in S1 and from 1101 to 777 ml/min in S2). Both patients experienced later AVF failure after the last follow-up of the current work. On the contrary, patients D1 and D2 experienced excessive vein dilatation, with diameters increasing to more than twice the recommended clinical guideline size of 6 mm. D1 exhibited uniform vein dilatation (up to 14.8 mm in diameter) starting at anastomosis, whereas for D2, the diameter of the juxta-anastomotic segment remained unchanged but the patient developed significant dilatation at the vein curvature (up to 13.1 mm in diameter). The increase in vessel diameter was accompanied by an increase in brachial blood flow volume in both patients (from 577 ml/min to 1545 ml/min in D1 and from 403 ml/min to 801 ml/min in D2). Patient D1 continued on hemodialysis and later underwent a kidney transplantation. Patient D2 experienced AVF closure due to a thrombus caused by hypotension a few weeks after the last follow-up, and AVF patency was restored using a Fogarty procedure. After the transplant, this AVF experienced further dilatation and an increase in blood flow and was closed due to a high risk of cardiac overload.

### High-Frequency Wall Vibrations and AVF Outcome

**Figure 4:**
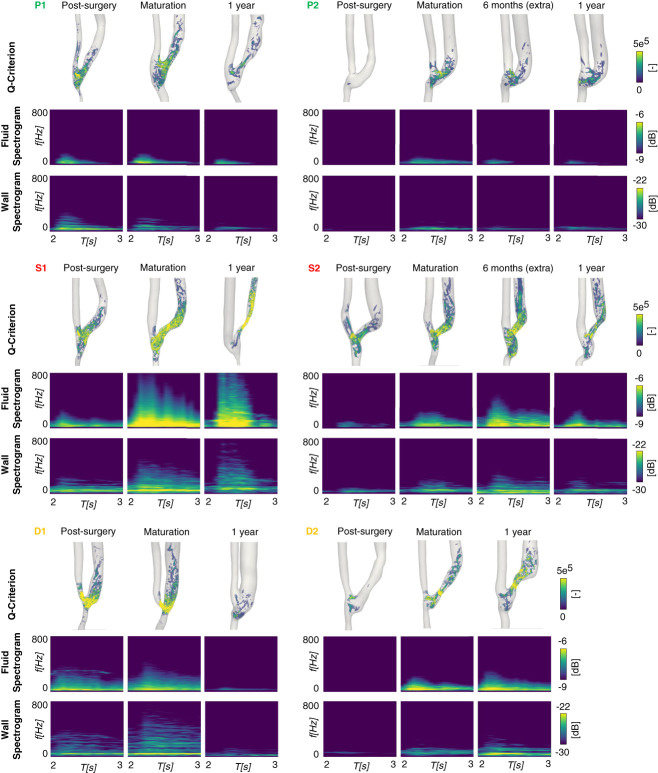
**Effect of transitional flow on high-frequency velocity fluctuations and vascular wall vibrations in the six AVFs under study, at different time points.** For each patient, the first row displays vortex visualization at peak systole using the Q-criterion, the second row shows the fluid velocity spectrograms, and the third row presents the wall displacement spectrograms. Spectrograms were extracted in the juxta-anastomotic vein region. In the spectrograms, dark blue represents low spectral power, while yellow indicates high spectral power.

**Figure 5:**
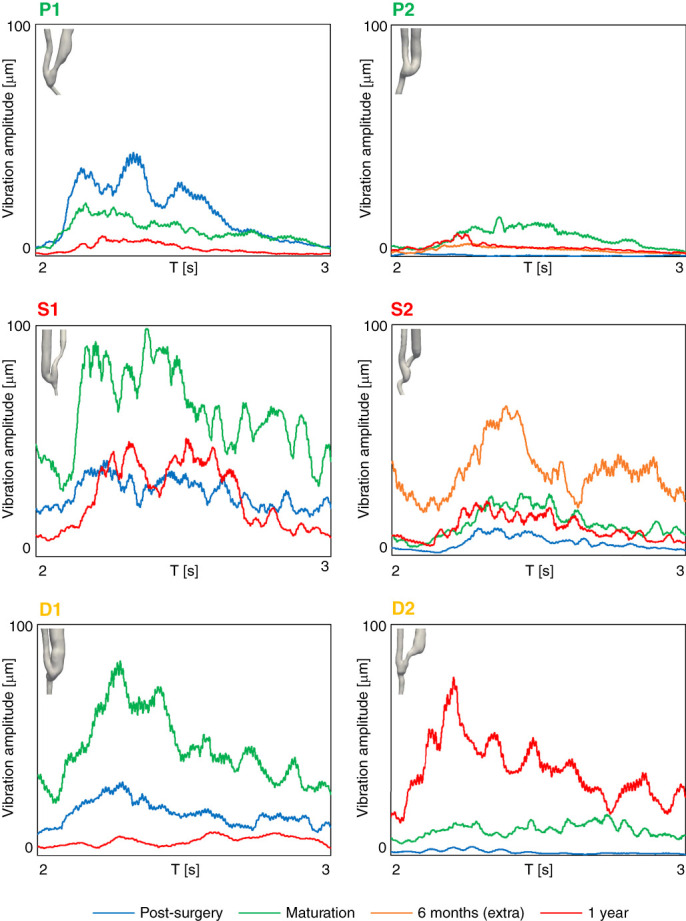
**Temporal evolution of wall vibration amplitudes throughout a cardiac cycle for the six AVFs under study.** The 99^th^ spatial percentile of vibration amplitude is used as the representative measure for vibrations. The 3D models in the figure are representative of AVF clinical outcome of the AVF.

Patients P1 and P2, who experienced good patency over time, exhibited minor flow instabilities at the anastomosis and within the JAV. As a result, the fluid velocity and wall displacement spectrograms (see Figure 4) show negligible vibrations, with frequencies and amplitudes progressively decreasing over time. Consistently, the pointwise vibration amplitudes shown in Figure 5 remained low and gradually decreased over time as well.

By contrast, patients who developed complications exhibited higher vibration frequencies and amplitudes. Stenotic patients S1 and S2 exhibited elevated Q-Criterion levels in the JAV segment, indicating a strong deviation from laminar to transitional flow. This altered flow pattern resulted in high-frequency fluctuations in blood flow velocity and vein wall displacement, as illustrated in the spectrograms (see Figure 4). Patient S1 demonstrated the highest frequency fluctuations after AVF maturation, which persisted up to the 1-year follow-up. These fluctuations reached up to 800 Hz, with amplitudes ranging from −8 to −6 dB at the systolic peak for fluid velocity and up to 700 Hz with amplitudes between −28 and −22 dB for wall displacement. Similarly, Patient S2 exhibited velocity fluctuations up to 500 Hz and wall displacement fluctuations up to 350 Hz at 6 months, which decreased following the development of stenosis at 1 year. Notably, both patients exhibited two distinct high-frequency bands in the displacement spectrogram before the onset of stenosis, with the lower-frequency band showing the highest vibration amplitude: 55–65 Hz and around 100 Hz for S1 and 45–50 Hz and 70–75 Hz for S2. After stenosis developed, these bands persisted but shifted to slightly higher frequencies and lower amplitudes. This pattern is further confirmed by the pointwise vibration data (see Figure 5), which shows the highest amplitudes before stenosis onset (S1: peak=100 *μ*m, cycle-average = 64 *μ*m; S2: peak=65 *μ*m, cycle-average = 36 *μ*m), followed by a decrease after stenosis development (S1: peak=52 *μ*m, cycle-average = 25 *μ*m; S2: peak=24 *μ*m, cycle-average = 12 *μ*m), although the values remained relatively elevated.

As regards patients who experienced excessive venous dilatation, high-flow instabilities were already observed near the anastomosis in patient D1 after AVF creation. As a result, velocity fluctuations up to 450 Hz were detected immediately after AVF surgery, persisting throughout the maturation period (see Figure 4). Low-amplitude wall vibrations (around −28 dB) were observed up to 600 Hz, with a prominent frequency band (exceeding −22 dB) consistently detected at around 50 Hz during the maturation phase. Notably, this high-frequency content was suppressed following significant dilatation of the JAV at 1 year. Consistently, the highest pointwise vibration amplitudes were observed at the time of maturation (peak=84 *μ*m, cycle-average = 46 *μ*m) and became almost negligible after remodeling (see Figure 5). By contrast, Patient D2 exhibited a different progression. Initially, negligible flow instabilities and no vibrations were observed immediately after surgery. However, after AVF maturation, flow instabilities arose at the JAV curvature, leading to a gradual increase in both velocity and displacement frequencies, reaching up to 300 Hz at 1 year. As in the previous case, the most prominent vibration mode detected was at around 50 Hz. This trend, with increasing vibrations from post-surgery through maturation to the 1-year follow-up, was also reflected in the vibration amplitudes, with the highest values recorded at 1 year (peak=77 *μ*m, cycle-average = 37 *μ*m).

**Figure 6:**
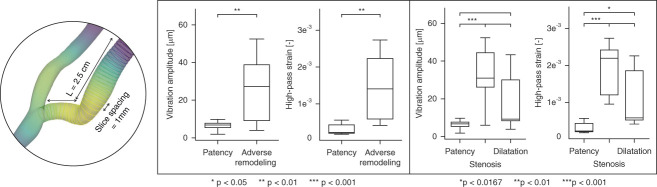
**Boxplots illustrate differences in the distributions of time-averaged vibration amplitude and high-pass strain across groups.** Cross-sectional slices from simulation results, extracted within the first 2.5 cm of the vein with 1 mm slice spacing, were used for statistical analysis. A generalized linear mixed-effects model was fitted, with Vibration amplitude and Strain as the response variables, Group (Patency, Stenosis, and Dilatation) as a fixed effect, and Patient as a random effect to account for repeated measures within individual patients. The analysis first combined all patients with adverse remodeling and then distinguished between the Stenosis and excessive Dilatation groups. For comparisons between the three groups, *P* values were adjusted using Bonferroni correction. The number of “*” symbols indicates the difference in levels of significance between groups.

The statistical analysis of the cross-sectional slices extracted at time point *t*−1, as shown in Figure 6, revealed that patients in the Patency group exhibited significantly lower variability in vibration amplitude and high-pass strain compared with the Stenosis and Dilatation groups, and markedly lower values that were more closely clustered around the median. The generalized linear mixed model on the cross-sectional slices revealed significant differences in time-averaged vibration amplitudes (6.6±2.0 *μ*m versus 22.5±5.8 *μ*m, *P* < 0.01) and high-pass strain ([0.30±0.10] 10^−3^ versus [1.30±0.35] 10^−3^, *P* < 0.01) between patients with proper patency and those with complications (Adverse Remodeling). Moreover, further distinguishing between Stenosis and extreme Dilatation, a significant difference in vibration amplitude was confirmed between the Patency and Stenosis groups (6.6±2.0 *μ*m versus 31.7±9.7 *μ*m, *P* < 0.001), while the difference between Patency and Dilatation was NS after Bonferroni correction (6.6±2.0 *μ*m versus 15.9±4.8 *μ*m, *P* > 0.0167). Regarding high-pass strain, a significant difference was confirmed between the Patency and Stenosis groups ([0.30±0.10] 10^−3^ versus [1.68±0.58] 10^−3^, *P* < 0.01) as well as between the Patency and Dilatation groups ([0.30±0.10] 10^−3^ versus [1.00±0.35] 10^−3^, *P*<0.0167).

### Relation between Conventional Hemodynamics and Vibrations

**Figure 7:**
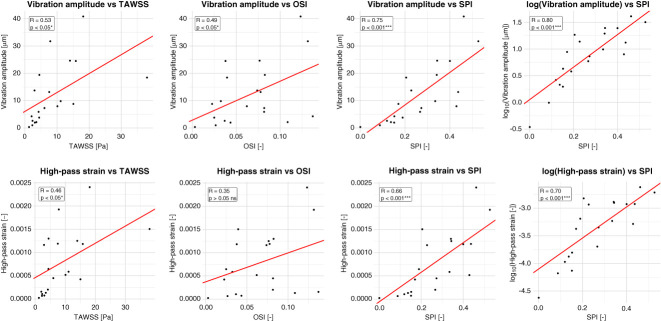
**Correlations between hemodynamic parameters (TAWSS, OSI, SPI) and vibrations (vibration amplitude and high-pass strain).** Each panel shows the linear relationship estimated using mixed-effects models (red line) with patient as a random factor. *R* values represent conditional correlation coefficients, and *P* values indicate statistical significance levels (*P* < 0.05*, *P* < 0.01**, *P* < 0.001***). Additional panels display SPI correlations with log-transformed variables, showing slightly improved fits. OSI, oscillatory shear index; SPI, spectral power index; TAWSS, time-averaged WSS.

The correlation analysis between hemodynamics and vibrations is presented in Figure 7. For vibration amplitude, only SPI exhibited a strong and highly significant correlation (*R*=0.75, *P* < 0.001), whereas TAWSS and OSI showed low-to-moderate correlations (*R*=0.53, *P* < 0.05; *R*=0.49, *P* < 0.05, respectively). For high-pass strain, TAWSS exhibited a low-to-moderate significant correlation (*R*=0.46, *P* < 0.05), OSI was not significantly associated (*R*=0.35, *P* > 0.05), and SPI again showed a strong and highly significant correlation (*R*=0.66, *P* < 0.001). When applying a logarithmic transformation to vibration metrics, the correlations with SPI slightly improved (*R*=0.80 and *R*=0.70; *P* < 0.001). Correlations with TAWSS and OSI remained unchanged. For clarity, only the SPI-related plots are shown in the logarithmic scale.

## Discussion

Here, we have presented a longitudinal FSI investigation involving six patients with native radio-cephalic AVF.

We found that AVFs that maintained proper patency over time exhibited negligible high-frequency content in the fluid velocity and wall displacement spectrograms throughout the whole investigation period. By contrast, vascular wall vibrations at high frequencies were consistently present in all AVFs that developed complications, whether due to stenosis development or excessive dilatation. Specifically, high-frequency wall vibrations were prominent at the timepoint before stenosis or excessive dilatation onset, suggesting a possible relation with adverse vascular remodeling. Among the AVFs with complications, the spectrograms of those that developed stenosis exhibited distinctive features compared with those with excessive venous dilatation. Specifically, patients who developed intimal hyperplasia exhibited two prominent narrow bands (in the 45–65 Hz and 70–100 Hz ranges) in the wall displacement spectrogram before inward remodeling occurred. These bands further increased in frequency and decreased in amplitude after the onset of stenosis, likely due to vessel narrowing and blood flow reduction, respectively. By contrast, patients who experienced excessive dilatation exhibited a single, prominent narrow band at a lower frequency (around 50 Hz) before remodeling. This suggests that different frequency levels may be associated with distinct types of vascular remodeling.

The evolution of vibration amplitudes in AVFs over time and the relation with clinical outcomes also deserves consideration. Patients S1 and S2 exhibited increased vibration amplitudes before the formation of stenosis. These amplitudes were damped after vascular remodeling, which can likely be attributed to the laminarization of flow and the reduction in blood flow volume observed following lumen occlusion.^35^ However, the amplitudes detected at 1 year were not completely damped and remained relatively high, suggesting that inward remodeling may have continued over time. Notably, both patients experienced AVF failure a few months after the last follow-up of this investigation, as verified from their medical records. Although we acknowledge that medical images were available only for the intermediate stenosis stage, the persistence of elevated vibration amplitudes may indicate a progressive pathologic process. Patient D1 also experienced an attenuation of vibration amplitudes after remodeling, although this occurred following significant dilatation of the JAV. Unlike stenotic AVFs, in this one—in which blood flow remained markedly high—the attenuation of vibrations could be attributed to a substantial decrease in blood velocity, resulting from extreme dilatation. After the current investigation ended, Patient D1 underwent transplantation, and the observed attenuation of vibrations suggests that the JAV may not have experienced further dilatation. No complications with the AVF were reported in his clinical records after transplantation. By contrast, Patient D2 exhibited increasing AVF vibration amplitudes from maturation to the last follow-up, indicating that dilatation near the JAV curvature may have continued to progress over time. Following the transplant, received a few months after this investigation ended, the AVF experienced further dilatation, as expected, and was eventually closed due to the risk of cardiac overload. Thus, the analysis of AVF vibration amplitude evolution over time suggests that higher vibration amplitudes may indicate future prominent vascular remodeling, while this amplitude is reduced after remodeling occurs.

These observations were supported by statistical analysis. Indeed, we found that AVFs in the Adverse Remodeling group exhibited significantly higher vibration amplitudes and strain before remodeling, compared with those in the Patency group. Significant differences in high-pass strain were observed between the Patency and Stenosis groups, as well as between the Patency and Dilatation groups. In addition, a significantly higher vibration amplitude was observed in stenotic patients compared with those with proper patency. However, the difference between Patency and Dilatation was not statistically significant after Bonferroni correction, suggesting that while increased vibration amplitudes may be linked to excessive dilatation, the relationship appears to be less pronounced compared with that in stenotic AVFs. Thus, the results suggest that specific vibration frequencies and amplitudes are associated with distinct types of vascular remodeling, despite the limited number of patients involved.

The correlation analysis between vibrations and WSS parameters showed that only SPI exhibited a strong and significant association. This finding is plausible, since vascular vibrations are flow-induced, and is consistent with the definition of SPI, which was specifically designed to describe turbulent-like flows and incorporates frequency-based contributions in its formulation.^42^ By contrast, the most commonly used TAWSS and OSI showed weaker correlations, indicating limited association with wall vibrations.

The principal limitation of this work is its small sample size, dictated by data acquisition and computational constraints. Although considerable for *in silico* studies, the number of cases remains limited from a clinical perspective, potentially affecting the generalizability of the findings. Moreover, owing to patient heterogeneity, we could not assess the impact of comorbidities or baseline vessel sizes on the outcomes. Future studies including larger cohorts would enable more robust statistical analyses and help validate the present preliminary observations.

Moreover, it should be noted that none of the AVFs in this cohort experienced early maturation failure; therefore, no association between vibration patterns and early failure could be established.

Finally, while our simulations reproduced vibration amplitudes consistent with those measured in experimental setups,^43,44^ we acknowledge the need for future rigorous validation of the FSI outcomes through *in vivo* or *in vitro* studies that replicate patient-specific AVF geometries.

From a mechanobiologic perspective, after decades of studies attempting to link WSS on the endothelium to AVF failure,^26^ we have demonstrated, for the first time, that flow-induced high-frequency vibrations may play a role in the process of vascular wall remodeling in AVFs. Although most experimental studies have focused on stable or oscillatory flow on endothelial cells,^45,46^ our findings align with the little evidence that has been reported in the literature showing that high-frequency mechanical stimuli can influence cellular phenotypes and gene expression in both endothelial and vascular smooth muscle cells, leading to structural and functional changes.^32^ These changes include alterations in cytoskeletal organization,^47^ disruption of the internal elastic lamina,^48–50^ cell proliferation,^50–52^ the production of signaling factors, and the activation of pathways related to inflammation, oxidative stress, and vascular dysfunction,^53–56^ all of which could have contributed to the adverse vascular remodeling of the AVF we observed in this work. Key effectors and signaling pathways, such as extracellular signal-regulated kinase 1/2,^57^ that lead to intimal thickening and recruitment of inflammatory cells to damaged vessel sites, Syn4, vascular endothelial growth factor, KLF2,^58^ ICAM-1,^48^ and NFATc3^49^ have been identified in responding to high-frequency vibrations. Furthermore, high-frequency vibrations have been found to influence muscle atrophy pathways,^47^ potentially leading to hypertrophic growth of skeletal muscle which could explain stenosis development occurring in vascular diseases.

The research presented in this work holds significant translational value. Indeed, if the influence of high-frequency mechanical stimuli on AVF failure is confirmed in larger cohorts, this could have significant implications for improving clinical practice and AVF surveillance, as vibrations may serve as early indicators of complications. Notably, the vibrations detected by FSI simulations in our work, as described here, can easily be felt in the arms of patients with AVF. Some nurses and clinicians currently monitor AVF function based on qualitative assessments of these skin vibrations, the AVF thrill, and by listening to associated sounds with a stethoscope.^2^ However, these methods are subjective and rely heavily on the clinician's experience and judgment, underscoring the need for more objective criteria. In this regard, defining specific vibration frequency and amplitude levels through FSI simulations could provide critical insights for clinical practice, identifying thresholds that could serve as biomarkers for different types of vascular remodeling. In the future, if confirmed, the outcomes of this research could pave the way for a rapid, noninvasive, and cost-effective device capable of quantifying vessel vibration amplitudes and related frequencies. Such a device could provide continuous and objective monitoring of AVFs, significantly enhancing surveillance and allowing for the timely identification of patients at risk of developing complications. Moreover, this research could inspire the design of novel devices that would aim to mechanically limit AVF wall vibrations, and promote surgical techniques for creating AVF configurations that minimize the onset of wall vibrations.

To conclude, this work highlighted the relationship between flow-induced vascular wall vibrations and AVF remodeling and failure. Our preliminary findings revealed that specific vibration frequencies and amplitude levels were associated with distinct types of vascular remodeling, suggesting their potential role in the mechanobiology of vascular cells and as biomarkers for AVF surveillance. Future studies involving larger cohorts will be essential to confirm these findings.

## Supplementary Material

## Data Availability

Original data generated for the study will be made available upon reasonable request to the corresponding author. Data Type: Health Care Data; Image Data; Raw Data/Source Data; Software Executable Code. Reason for Restricted Access: The data of MRI, DUS examinations, and postprocessing results supporting the findings of this work are fully anonymized and openly available in the Zenodo repository at 10.5281/zenodo.14754994, following a specific request to the authors. The code used for FSI simulations is available in VaSP github repository at https://github.com/KVSlab/VaSP.

## Declarative Statements

This study is exempt from the Institutional Review Board or Ethics Committee approval, and we have provided the reason for exemption. Six patients were followed in routine clinical practice at ASST Papa Giovanni XXIII. All clinical data were fully anonymized and analyzed retrospectively; therefore, no patient consent was required.

## Supplemental Material

Supplemental Methods. Fluid-structure interaction pipeline.
