# Supplementary material for: The Role of High-Frequency Wall Vibrations in Adverse Vascular Remodeling of Arteriovenous Fistula for Hemodialysis
Source: Kidney360. 2026 Jan 14;7(5):1104–17. doi: 10.34067/KID.0000001112 (PMC13229424; doi:10.34067/KID.0000001112)
Supplement: Supplementary file 2 [file kidney360-7-1104-s002.pdf]

Supplemental material to

The role of high-frequency wall vibrations in adverse vascular remodeling of arteriovenous fistula for hemodialysis

Luca Soliveri, Sofia Poloni, Paolo Brambilla, Simona Zerbi, Giulia Cabrini, Anna Caroli, Andrea Remuzzi, Kristian Valen-Sendstad, Michela Bozzetto

Kidney360

Table of contents

Supplemental Methods. Fluid-structure interaction pipeline

Supplemental Methods

Fluid-structure interaction pipeline

A detailed description of the fluid-structure interaction (FSI) pipeline and its implementation is provided in our recent publication[1].

Patient-specific 3D surface models of the arteriovenous fistulas (AVFs) were reconstructed based on magnetic resonance imaging (MRI) scans at each time point. Meshes consisting of approximately 200,000 tetrahedral elements (140,000 for the fluid and 60,000 for the solid domain) were generated, building upon a previous mesh refinement study[1], with two boundary layers for both the fluid and solid domains, using an in-house Python script based on the VMTK libraries.

At 3 days post-surgery, the radial artery was modeled to be both thicker and stiffer than the cephalic vein[2]. Specifically, a radial artery thickness of 0.3 mm was assigned, while the cephalic vein was initially modeled with a thickness of 0.2 mm [3,4]. The vascular wall was modeled using a 3-term Mooney-Rivlin non-linear hyperelastic soft tissue model, capturing the distinct mechanical behaviors of the radial artery and cephalic vein at the time of AVF creation[1]. Starting at 40 days, the vein thickness was increased to 0.4 mm and the cephalic vein was simulated using the same coefficients as the radial artery, to reflect vascular wall thickening and stiffening during maturation[5]. In the absence of patient-specific measurements, wall thickness increase was estimated from literature data, reporting an average cephalic vein wall thickness of 0.34 ± 0.12 mm [4].

High-fidelity FSI simulations were conducted using the open-source solver turtleFSI[6]. This fully coupled, monolithic solver provides second-order accurate solutions in time, using quadratic Taylor–Hood ($\mathbb{P}2$-$\mathbb{P}1$) elements for the velocity-pressure field and quadratic ($\mathbb{P}2$) elements for the solid deformation, achieving $\mathbb{P} + 1$ accuracy (L2) in space. With a mesh size of approximately 200,000 cells, this setup corresponds to at least 1.6 million linear elements, resulting in an effective average node spacing of 0.1825 mm. In the monolithic formulation, the fluid and solid equations are solved

simultaneously by combining all governing equations into a single variational form. This approach employs a shifted Crank–Nicholson scheme (i.e., $\Theta = 0.5 + dt$) and Newton's method to solve the non-linear parts of the governing equations. The absolute and residual tolerances of the Newton solver were both set to 10^{-7}. For mesh lifting, a second-order Laplace equation, suitable for small deformations, was used. A small simulation timestep of 0.1 ms (equivalent to 10,000 timesteps per second) was selected to accurately capture potential high-frequency fluctuations in velocity and pressure[7], ensuring numerical convergence.

The extremities of the model were assumed to be rigid and fixed in space, while a sphere with a radius of 2.5 cm centered at the anastomosis defined the compliant region of interest within the computational domain[8–10]. During image acquisition, the vascular wall was in a state of stress equilibrium with the hemodynamic pressure. To replicate this equilibrium state in the simulations, the AVF model was iteratively shrunk and inflated using cycle-averaged pressure to achieve a close match with the medical images following pressure initialization[1].

Patient-specific flow waveforms obtained from Doppler ultrasound (DUS) measurements were imposed at the inlet of the proximal and distal artery, while a zero-pressure (do-nothing) condition was applied at the outlet. Due to the unavailability of direct invasive measurements of outflow vein pressure, we relied on the previously verified and validated AVF.SIM 1D solver[11] to compute time-varying pressure curves, which were applied as a stress at the fluid–solid interface. The solver takes as input patient-specific data, such as vessel diameters and blood flow volumes, and provides plausible pressure boundary conditions as output.

Three complete cardiac cycles were simulated, resulting in a total simulation time of 3.0 physical seconds. The first cycle was used to initialize velocity and pressure with a sigmoid function, smoothly ramping up to physiological conditions. Velocity was initialized during the interval [0–0.2] seconds, while pressure initialization started after the velocity ramp-up at 0.05 seconds, to prevent flow reversal in the vein due to the increased volume of the computational domain[1].

Blood was modeled as a Newtonian fluid with a density of 1.025 g/cm³ and a viscosity of $3.5 \cdot 10^{-6}$ m²/s. The viscoelastic effect of perivascular tissue was mimicked with Robin boundary conditions, with an elastic parameter of $k_s = 10^5\ N/m^3$ and viscoelastic parameter of $c_s = 10\ (N \cdot s)/m^3$, based on calibrations from our previous study[1].

The approximate computing time to solve one physical second ranged from 32 to 72 hours, using a compute node with 40 Intel Xeon Gold 6138 2.0 GHz cores on the Saga HPC cluster.

Only the third cycle was used for post-processing the results to minimize the effects of artificial initial conditions. The Q-criterion, a commonly used method for visualizing vortices and flow instabilities, was calculated as

$$\boldsymbol{Q} = \frac{1}{2} ||\boldsymbol{\Omega}||^2 - ||\boldsymbol{S}||^2$$

and used to identify areas of unstable flow. Specifically, $\boldsymbol{\Omega}$ is the rotation rate or vorticity tensor, while $\boldsymbol{S}$ denotes the strain rate tensor. Here, $\boldsymbol{S}$ and $\boldsymbol{\Omega}$ represent the symmetric and antisymmetric parts of the velocity gradient tensor, respectively. Additionally, the Green–Lagrange strain $\boldsymbol{E}$ was calculated as

$$\boldsymbol{E} = \frac{1}{2}(\boldsymbol{C} - \boldsymbol{I})$$

where $\boldsymbol{C}$ represents the right Cauchy–Green deformation tensor and $\boldsymbol{I}$ the identity tensor. The tensor $\boldsymbol{C}$ is obtained from the deformation gradient tensor $\boldsymbol{F}$, which describes the change in position of material points in a deforming body, as $\boldsymbol{F}^T\boldsymbol{F}$.

A frequency of 25 Hz was used as the cutoff threshold for high-pass filtering, consistent with other work on flow instability in vascular lesions[9,12]. A high-pass filter was applied to isolate the vibrations of the vascular wall. Displacements exceeding 25 Hz were categorized as vibrations, while those below were classified as inflation due to pressure pulsation[9]. Since instantaneous values of wall displacement, fluid velocity, or strain alone cannot fully describe the magnitude or location of high-frequency fluctuations, a method to calculate the moving average amplitude for these quantities[9] was

proposed. First the *X*, *Y*, and *Z* components of the wall displacement and fluid velocity vector, and all 9 components of the Green–Lagrange strain tensor at every node were high-pass filtered. Then, the windowed root mean squared amplitude of each component was calculated with a rectangular temporal window length of 250 timesteps, resulting in a velocity and displacement amplitude vector and strain amplitude tensor. For velocity and displacement, the magnitude of the amplitude vector was calculated. For the Green–Lagrange strain, the maximum principal value of the amplitude tensor at each node was used to represent the spatial distribution of high-frequency fluctuations. The 99th percentile spatial value was plotted as representative for vibration amplitude.

Spectrograms were generated to show the evolution of high-frequency content in velocity and displacement signals over the cardiac cycle, following methods from Natarajan et al.[13] and Bruneau et al.[9]. A short-time Fourier transform was used for the temporal window of interest (1 cycle, total time of 1.0 s, or 10,000 timesteps in total), with 75% windows overlapping. To obtain a representative spectrogram for the juxta-anastomotic vein, individual spectrograms of the high-pass-filtered velocity and displacement magnitude were calculated at each node in that region. Then, the average of the nodal magnitude spectrograms was taken as a representative for the whole region of interest. Power spectrum scaling was applied to the spectrograms (i.e., the units of the spectrogram were the squared units of the input), and the spectrogram plots were log-scaled.

References

1. Soliveri L, Bruneau D, Ring J, Bozzetto M, Remuzzi A, Valen-Sendstad K. Toward a physiological model of vascular wall vibrations in the arteriovenous fistula. *Biomech Model Mechanobiol*. 2024;23(5):1741-1755. doi:10.1007/s10237-024-01865-z

2. Marcinno' F, Vergara C, Giovannacci L, Quarteroni A, Prouse G. Computational fluid-structure interaction analysis of the end-to-side radio-cephalic arteriovenous fistula. *Computer Methods and Programs in Biomedicine*. Published online April 2024:108146. doi:10.1016/j.cmpb.2024.108146

3. Corpataux JM. Low-pressure environment and remodelling of the forearm vein in Brescia-Cimino haemodialysis access. *Nephrology Dialysis Transplantation*. 2002;17(6):1057-1062. doi:10.1093/ndt/17.6.1057

4. Lee T, Chauhan V, Krishnamoorthy M, et al. Severe venous neointimal hyperplasia prior to dialysis access surgery. *Nephrology Dialysis Transplantation*. 2011;26(7):2264-2270. doi:10.1093/ndt/gfq733

5. Remuzzi A, Bozzetto M. Biological and Physical Factors Involved in the Maturation of Arteriovenous Fistula for Hemodialysis. *Cardiovasc Eng Tech*. 2017;8(3):273-279. doi:10.1007/s13239-017-0323-0

6. Bergersen A, Slyngstad A, Gjertsen S, Souche A, Valen-Sendstad K. turtleFSI: A Robust and Monolithic FEniCS-based Fluid-Structure Interaction Solver. *JOSS*. 2020;5(50):2089. doi:10.21105/joss.02089

7. Khan MO, Valen-Sendstad K, Steinman DA. Narrowing the Expertise Gap for Predicting Intracranial Aneurysm Hemodynamics: Impact of Solver Numerics versus Mesh and Time-Step Resolution. *American Journal of Neuroradiology*. 2015;36(7):1310-1316. doi:10.3174/ajnr.A4263

8. Souche A, Valen-Sendstad K. High-fidelity fluid structure interaction simulations of turbulent-like aneurysm flows reveals high-frequency narrowband wall vibrations: A stimulus of mechanobiological relevance? *Journal of Biomechanics*. 2022;145:111369. doi:10.1016/j.jbiomech.2022.111369

9. Bruneau DA, Steinman DA, Valen-Sendstad K. Understanding intracranial aneurysm sounds via high-fidelity fluid-structure-interaction modelling. *Commun Med*. 2023;3(1):163. doi:10.1038/s43856-023-00396-5

10. Bozzetto M, Remuzzi A, Valen-Sendstad K. Flow-induced high frequency vascular wall vibrations in an arteriovenous fistula: a specific stimulus for stenosis development? *Phys Eng Sci Med*. 2024;47(1):187-197. doi:10.1007/s13246-023-01355-z

11. Bozzetto M, Poloni S, Caroli A, et al. The use of AVF.SIM system for the surgical planning of arteriovenous fistulae in routine clinical practice. *J Vasc Access*. Published online January 6, 2022:112972982110626. doi:10.1177/11297298211062695

12. Khan MO, Toro Arana V, Najafi M, et al. On the prevalence of flow instabilities from high-fidelity computational fluid dynamics of intracranial bifurcation aneurysms. *Journal of Biomechanics*. 2021;127:110683. doi:10.1016/j.jbiomech.2021.110683

13. Natarajan T, MacDonald DE, Najafi M, Khan MO, Steinman DA. On the spectrographic representation of cardiovascular flow instabilities. *Journal of Biomechanics*. 2020;110:109977. doi:10.1016/j.jbiomech.2020.109977
